# Intrathecal Morphine Injection for Postoperative Analgesia Following Gender-Affirming Pelvic Surgery: A Retrospective Case-Control Study

**DOI:** 10.7759/cureus.36748

**Published:** 2023-03-27

**Authors:** Matthew W Swisher, Isabella M Dolendo, Jacklynn F Sztain, Brenton S Alexander, Paige S Tsuda, Jennifer T Anger, Engy T Said

**Affiliations:** 1 Anesthesiology, University of California San Diego, La Jolla, USA; 2 Urology, University of California San Diego, La Jolla, USA

**Keywords:** gender-affirming surgery, neuraxial analgesia, multimodal pain management, pelvic surgery, acute postoperative pain, intrathecal morphine therapy

## Abstract

Background

Gender-affirming pelvic surgery (GAPS) can be associated with significant postoperative pelvic pain. Given the lack of available peripheral nerve blocks to the perineum, intrathecal morphine (ITM) injection could offer a potent analgesic modality for this patient population. No prior studies to date have been performed examining the analgesic effects of intrathecal morphine for these patients.

Methods

This retrospective case-control study aims to understand the postoperative analgesic effects of intrathecal morphine for these patients with a historical comparison group of patients who did not receive intrathecal morphine.

Results

Fourteen patients presented for gender-affirming pelvic surgery over an eight-month period at a single institution and were offered intrathecal morphine for postoperative analgesia. Their analgesic results were compared to a similar historical group of 13 patients who were not offered or declined intrathecal morphine.

Conclusions

Intrathecal morphine injection is a potent analgesic modality for patients presenting for gender-affirming pelvic surgery.

## Introduction

The transgender and nonbinary (TGNB) population in the United States is rapidly growing, and gender-affirming pelvic surgery (GAPS) is becoming more accessible as new legislation broadens insurance coverage. A meta-analysis of national surveys from 2007 to 2015 showed that the TGNB population increased during this time period and estimated almost 1 million TGNB Americans in 2016 [1]. The prevalence of self-reported transgender identity ranges from 0.5% to 1.3% [2]. Between 2015 and 2018, transmasculine procedures increased by 392%, while transfeminine procedures increased by 109% [3]. This increase is likely to continue as more institutions develop programs dedicated to gender-affirming care and more surgeons receive training in these procedures.

With this increase in gender-affirming pelvic surgery, anesthesiologists are also challenged with managing these patients with unique biological, hormonal, and psychosocial needs [4,5]. Anesthesiologists must be aware of factors including the effects of hormone therapy on the perioperative period and potential airway complications due to masculinization or feminization procedures [6,7].

Postoperative pain control remains an important target to improve outcomes of GAPS. Transgender patients have higher rates of certain psychological comorbidities including anxiety and depression that play a role in acute pain management [8]. Additionally, transgender patients have higher rates of chronic pain conditions, which must be considered when developing a perioperative pain regimen [9].

Intrathecal opioids offer short-term analgesia at relatively low doses since the drug is released into the subarachnoid space and is directly delivered to opioid receptors and ion channels. Intrathecal opioid administration has been shown to be effective for postoperative analgesia for many different types of surgeries including obstetric, abdominal, and cardiothoracic procedures [10-13]. However, there is a relative paucity of research on intrathecal opioid use in pelvic surgeries. Rebel et al. retrospectively examined analgesia in patients undergoing non-GAPS and found that those who received intrathecal opioids had better postoperative pain scores and decreased opioid consumption compared to patients who received systemic analgesia with opioids [14].

Postoperative analgesia following GAPS can be challenging, and optimal postoperative analgesia regimens have yet to be established for this patient population [4]. While combined epidural and general anesthesia has been shown to provide superior analgesia for patients undergoing penile inversion vaginoplasty compared to general anesthesia alone [15], to date, no studies have evaluated the effectiveness of intrathecal opioids in GAPS. We sought to retrospectively examine the effect of intrathecal morphine (ITM) on pain scores and opioid consumption in patients undergoing GAPS.

## Materials and methods

The Institutional Review Board of the University of California San Diego, La Jolla, California, approved this retrospective case-control study (protocol number: 200392) and the manual collection of anonymized data from the institution's electronic medical record. Informed consent was waived for the study. This study followed the Enhancing the QUAlity and Transparency Of health Research (EQUATOR) reporting guidelines for case-control studies (Strengthening the Reporting of Observational Studies in Epidemiology {STROBE}).

Between April 2022 and December 2022, 15 patients presented for gender-affirming pelvic surgery by one surgeon (JTA) and were offered ITM in the preoperative area for postoperative analgesia. Fourteen patients agreed to ITM and provided informed consent for the procedure. Another 12 patients were not offered ITM since the offering at our institution had not yet been standardized for this surgical population. The one patient who declined ITM and the 12 patients who were not offered ITM represented our historical control group.

After informed consent was provided in the preoperative area, the patients were positioned in the sitting position, and standard monitors were applied. After a preprocedural time-out was performed, mild sedation was provided with intravenous midazolam and/or fentanyl based on the patient's wishes. The lower lumbar area was prepped with 10% povidone-iodine solution and draped under sterile conditions. After skin infiltration was performed with 1% lidocaine, an introducer was placed, and a 25-gauge Whitacre spinal needle was advanced into the intrathecal space between the second and fifth lumbar vertebrae. After the confirmation of cerebrospinal fluid, 250-300 μg of preservative-free morphine was then injected, and the needle was withdrawn (13 patients received 300 μg; one patient received 250 μg).

The patients subsequently underwent general anesthesia in the operating room, and the ITM group was followed by our acute pain service to assist with pain management and multimodal analgesia typically with opioids, acetaminophen, gabapentinoids, and non-steroidal anti-inflammatory drugs. Postoperative respiratory monitoring for ITM patients was performed per the American Society of Anesthesiologists guidelines [16].

The primary outcome for this study was opioid consumption measured in oral morphine equivalents (MEQ) in milligrams from the postoperative anesthesia care unit (PACU) until the morning of the postoperative day (POD) 2 between the two groups. Secondary outcomes included pain scores as expressed in numeric rating scale (NRS) (0-10), length of stay, and adverse events. Statistical analysis was performed comparing median values for each outcome using the Mann-Whitney test, and p<0.05 was considered statistically significant.

## Results

Over an eight-month period, 15 patients presented for GAPS and were offered ITM for postoperative analgesia. One of these patients declined ITM and was included with a historical control of 12 other patients who were not offered ITM. Table 1 details the patient demographics and surgical characteristics in both groups.

**Table 1:** Patient demographics and surgical characteristics Values are reported as median (interquartile) or number (percentage of subjects) ASA, American Society of Anesthesiologists; ITM, intrathecal morphine

	ITM Group (n=14)	Non-ITM Group (n=13)
Age	54 (30-60)	42 (33-50)
Gender identity: female	10 (71)	9 (69)
Gender identity: male	4 (29)	4 (31)
ASA class I	5 (36)	6 (46)
ASA class II	9 (64)	6 (46)
ASA class III	0 (0)	1 (8)
Patients taking preoperative opioids	2 (14)	1 (8)

Overall, two patients in the ITM group were on preoperative opioids compared to one patient in the non-ITM group. Of the specific types of GAPS performed, the most common procedures included vaginoplasties (penile inversion and robotic-assisted peritoneal flaps or colonic vaginoplasties) and vulvoplasties (shallow-depth) including penectomy/orchiectomy/labiaplasty/clitoroplasty, as well as metoidioplasties (with or without vaginectomy and/or hysterectomy).

**Figure 1:**
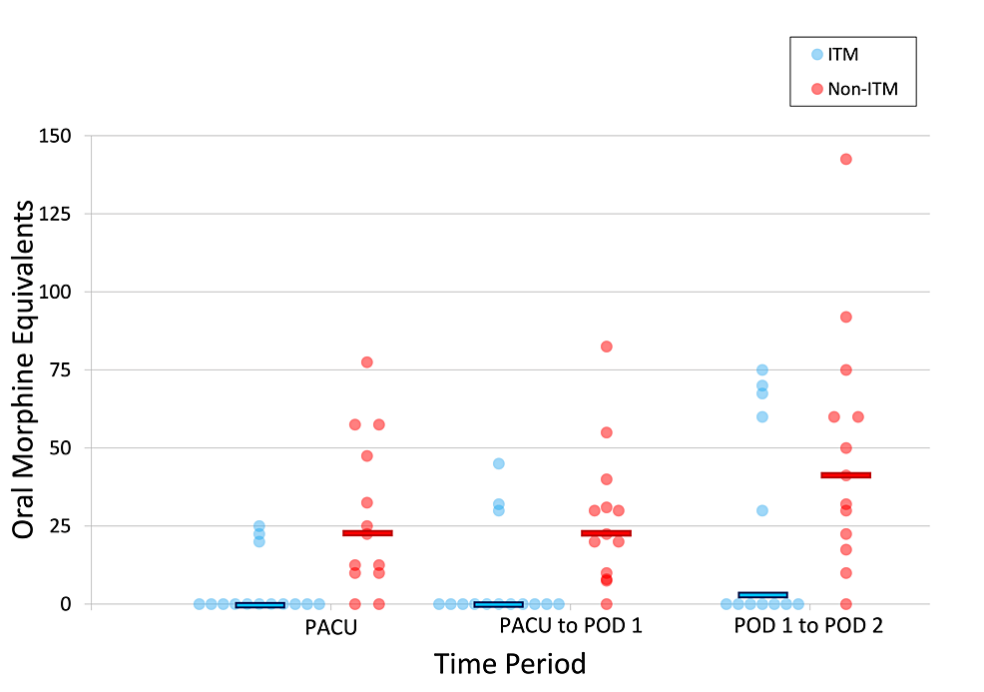
Median opioid consumption in oral morphine equivalents for the postoperative time periods between the two groups The horizontal bars represent the overall median for that time period PACU, postoperative anesthesia care unit; POD, postoperative day; ITM, intrathecal morphine

**Figure 2:**
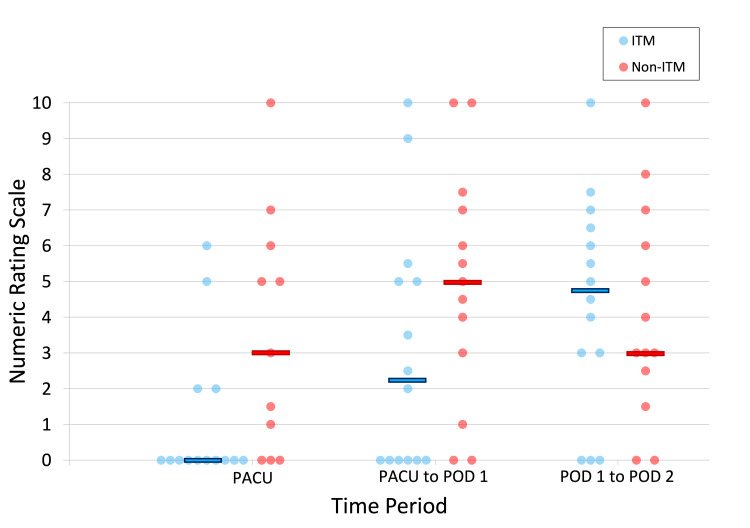
Median numeric rating scale pain scores (0-10) for the postoperative time periods between the two groups The horizontal bars represent the overall median for that time period PACU, postoperative anesthesia care unit; POD, postoperative day; ITM, intrathecal morphine

The patients in the ITM group had lower median opioid consumption in the PACU (0 mg versus 22.5 mg MEQ; p=0.001), from PACU to the morning of POD 1 (0 mg versus 22.5 mg MEQ; p=0.003), and the morning of POD 1 to morning of POD 2 (3.8 mg versus 41 mg MEQ; p=0.041) compared to the non-ITM group. In addition, the ITM group had lower median pain scores in the PACU (0 versus 3; p=0.029). While there was a trend toward lower pain scores from PACU to the morning of POD 1 in the ITM group (2.3 versus 5; p=0.125), this did not reach statistical significance. From the morning of POD 1 to POD 2, a trend toward higher median pain scores was seen in the ITM group (4.8 versus 3; p=0.656), but statistical significance was not achieved. Figures 1-2 show the distributions of opioid consumption and pain scores between the two groups.

In terms of opioid consumption, 11 patients in the ITM group (79%) required no opioids from PACU until the morning of POD 1 compared to only one patient in the non-ITM group (8%). From POD 1 to POD 2, seven patients in the ITM group (50%) required no opioids compared to only one patient in the non-ITM group (8%). At the time of discharge, five patients in the ITM group (36%) did not require a prescription for outpatient opioids compared to three patients in the non-ITM group (23%). The mean number of opioid tablets prescribed at the time of discharge was five for the ITM group and 10 for the non-ITM group.

A trend toward a lower overall median length of stay was observed in the ITM group compared to the non-ITM group (four days versus five days; p=0.326), but statistical significance was not achieved. Adverse events were seen in eight of the 14 ITM patients, which included low blood pressure or orthostasis in four patients, pruritis in two patients, and nausea/vomiting in three patients. For the non-ITM group, adverse events were seen in five of the 13 non-ITM patients, which included low blood pressure or orthostasis in two patients, nausea/vomiting in one patient, emergence delirium in one patient, and postoperative ileus in one patient. There were no recorded instances of delayed emergence, oversedation, apnea events, or respiratory depression in either group.

## Discussion

Preoperative ITM administration for postoperative analgesia following GAPS appears to be a safe and potent analgesic modality. This retrospective case-control study represents the first study examining the effects of ITM for postoperative analgesia in this increasing surgical population. For patients undergoing GAPS, ITM appears to result in decreased opioid consumption from PACU to POD 2 and decreased pain scores in the PACU. A high percentage of patients receiving ITM did not require any opioids in the early postoperative period compared to those that did not receive ITM, which suggests that ITM administration does confer a significant opioid-sparing effect postoperatively for these patients.

While not yet fully understood, pain management after GAPS can be challenging with limited options. In addition, persistent postsurgical pain has been found to be high following GAPS [17]. Since poorly controlled postoperative pain has been associated with the development of persistent postsurgical pain in other surgical populations, it is imperative to find optimal pain management modalities for patients undergoing GAPS [18].

Pharmacologic management for postoperative pain in GAPS patients includes the use of systemic opioids and non-opioid adjuncts such as non-steroidal anti-inflammatory drugs, gabapentinoids, ketamine, and lidocaine. Apart from the systemic pharmacologic management of postoperative pain, interventional options for pain control tend to be more limited for pelvic surgery. While commonly used for abdominal surgery, peripheral nerve blocks such as transversus abdominis plane blocks, paravertebral nerve blocks, or erector spinae plane blocks are less commonly used for pelvic surgery given the need for sacral dermatomal coverage and pudendal nerve involvement. Pudendal nerve blocks have not been widely adopted for major pelvic surgery [17]. Sacral erector spinae plane blocks have recently been described for GAPS, but larger studies outside of case reports are lacking [19]. In comparison to peripheral nerve blocks, local anesthetic-based neuraxial techniques, although effective, are limited in practice by the need for postoperative mobility in these patients. Opioid-based neuraxial techniques (such as intrathecal morphine) provide the opportunity for potent analgesia that cannot be achieved with peripheral nerve blocks without limiting mobility and ambulation.

ITM administration for GAPS represents a promising modality for postoperative analgesia given its proven efficacy in other surgical populations. The opioid-sparing effects of preoperative ITM in comparison to systemic analgesia or peripheral nerve blocks have been shown to be substantial in randomized studies for cardiac, colorectal, urologic, hepatobiliary, and abdominal vascular surgery, to name a few [13,20-24]. While commonly used for analgesia following obstetric surgery given the neuraxial nature of most obstetric anesthetics, ITM administration for non-obstetric surgery appears to be less common. Given the known opioid-sparing effects and safety profile of ITM combined with the limited peripheral nerve block options for pelvic surgery, ITM for GAPS patients represents a potent and safe modality for postoperative analgesia. This retrospective study represents the first study showing a significant opioid-sparing effect for ITM for GAPS patients.

Given its retrospective nature, this study does have several limitations. The association between ITM administration and decreased postoperative opioid consumption may be a result of other factors (such as acute pain service involvement in managing these patients' postoperative multimodal analgesia). The ITM group was followed closely by our acute pain service usually for 2-3 days, and multimodal analgesic regimen changes were made by our service with the goal of opioid minimization. For the non-ITM group, the postoperative regimen was instead managed by the surgical service. All patients in both groups received some form of postoperative multimodal analgesia, but the regimens might have differed between both groups. As such, differences in postoperative analgesic management may have contributed to the results. Ideally, a prospective, randomized study would be performed to examine causality and standardize the postoperative multimodal analgesic regimens between both groups. However, the recent adoption of ITM as standard of care for this patient population at our institution may preclude that type of study given the perceived clinical benefits. For the specific types of GAPS performed, there is a high degree of heterogeneity in the specific procedures being performed, which could have affected the results. We attempted to include all GAPS that would be predicted to result in at least moderate postoperative pain. Some surgeries (such as isolated minimally invasive hysterectomies) were not included since the degree of pain was not predicted to be as high as the included surgeries, and those surgeries may be more amenable to peripheral nerve blocks (i.e., transversus abdominis plane blocks).

## Conclusions

GAPS can be associated with difficult to control postoperative pain, and interventional options for pelvic pain after GAPS are limited. This retrospective study demonstrates that preoperative ITM decreases opioid consumption and early postoperative pain scores following GAPS without sacrificing mobility. ITM represents a potent analgesic option for this particular surgical population with a significant opioid-sparing effect early postoperatively. A definitive, highly powered randomized trial is warranted to further understand the treatment effect and safety profile.
